# *Enterococcus mundtii* Isolated from Slovak Raw Goat Milk and Its Bacteriocinogenic Potential

**DOI:** 10.3390/ijerph17249504

**Published:** 2020-12-18

**Authors:** Andrea Lauková, Valentína Focková, Monika Pogány Simonová

**Affiliations:** Centre of Biosciences of the Slovak Academy of Sciences, Institute of Animal Physiology, Šoltésovej 4-6, 040 01 Košice, Slovakia; fockova@saske.sk (V.F.); simonova@saske.sk (M.P.S.)

**Keywords:** goat milk, *Enterococcus mundtii*, bacteriocin activity, lactic acid bacteria

## Abstract

Enterococci are lactic acid bacteria. Most of them can adapt well to the food system due to their salt and acid-tolerance. Moreover, many enterococcal species have been found to produce antimicrobial substances of proteinaceous character, i.e., bacteriocins/enterocins. In this study, *Enterococcus mundtii* EM ML2/2 with bacteriocinogenic potential was identified in Slovak raw goat milk. This strain demonstrated inhibition activity against up to 36% of Gram-positive indicator bacteria, and in concentrated form the bacteriocin substance (pH 6.3) showed the highest inhibition activity (1600 AU/mL) against the principal indicator strain *E. avium* EA5. Semi-purified substance (SPS) EM ML2/2 produced inhibition activity up to 3200 AU/mL. Concentrated bacteriocin substance and SPS maintained active (inhibition activity up to 100 AU/mL) for three months under −20 °C storage conditions. The strain showed susceptible antibiotic profile, and it did not form biofilm. No production of damaging enzymes was noted. It was nonhemolytic, as well as DNase, and gelatinase-negative. It grew well in skim milk, and it was salt and acid-tolerant. The bacteriocin potential of *E. mundtii* species isolated from Slovak raw goat milk has not previously been detected, so this is an original contribution which may stimulate addtitional research and application studies.

## 1. Introduction

Enterococci form a special group of lactic acid bacteria (LAB) comprising both pathogenic and commensal species which are ubiquitous in the environment as gut symbionts [1]. Due to their salt and acid-tolerance, enterococci can adapt well to different food systems. They are involved in the fermentation process of some traditional cheeses and dry sausages to develop the required quality characteristics [2]. Moreover, several enterococcal species have been found to produce antimicrobial substances of proteinaceous character, i.e., bacteriocins/enterocins [3,4,5,6]. These substances also generate additional benefits in animals, such as stimulation of phagocytic activity (non-specific immunity parameter; *p* < 0.001), positively influencing morphometry in the jejunum of broiler rabbits (*p* < 0.0001) [7], or increasing their body weight [8] as well as reducing the incidence of *Eimeria* oocysts [9]. As it is, their use as beneficial (probiotic) bacteria has been reported many times in various environments/sources [7,8,9,10,11,12]. However, it is necessary all the time to bear in mind their safety concerns [13]. The European Food Safety Authority (EFSA) and the Advisory Committee on Novel Foods and Processes (ACNFP) and the Food Standards Agency recommend assessing enterococci based on consideration of individual strains and health-risk exclusion for their potential use as food additives and supplements [13,14]. Because enterococci are widespread bacteria, there is increasing interest in their more detailed testing, especially of those species which show potential for their beneficial/functional use in food production, e.g., *E. mundtii*. This species is a representative of the genus *Enterococcus* from the family Enterococcacae and phylum Firmicutes. Based on the 16S rRNA gene similarity, the species *E. mundtii* belongs in the E. faecium cluster [15]. Some strains of *E. mundtii* are able to produce bacteriocins, e.g., *E. mundtii* isolated from fish (in the Patagonia region of Argentina), which are active against *Listeria monocytogenes*, and *Shewanella putrefaciens* [16]. Another anti-listerial mundticin was reported by Feng et al. [17], the producer strain of which is *E. mundtii* CUGF08 from alfalfa sprouts.

Goat breeding has a long tradition in Slovakia, especially on small farms. It is often associated with agrotourism. Nowadays, people like to return to goat milk, and products from it are ever more frequently consumed. The originality of goat milk lies especially in its higher calcium content in comparison with cow milk. It also contains other minerals, and trace elements such as magnesium, sodium, phosphorus, copper, zinc, manganum and chromium. Besides the fact that goat milk is full of vitamins, it also contains short- and medium-chain fatty acids, e.g., butyric acid but mostly capronic, caprylic, caprinic acids, and also palmic acid, linolenic and arachidonic acids [18].

Because mostly *E. faecium* species can be detected in Slovak goat milk with probable bacteriocinogenic potential, but only one strain of *E. mundtii*, this study focuses on the *E. mundtii* species to study its antimicrobial (bacteriocinogenic) potential for possible application as bioprotection on goat farms and/or in goat milk products.

## 2. Materials and Methods

### 2.1. Sampling and Strain Isolation

Sampling of milk was done together with our colleagues directly on farms. A total of 53 raw goat milk samples collected from 283 goats in the regions of central and eastern Slovakia was transported in special refrigerated boxes to our laboratory. Fifty-one milks were taken from individual animals (51 goats), and two samples were pooled milks from two herds (132 goats).

After delivery, the samples were treated in the laboratory using the standard microbiological method according to the International Organization for Standardization (ISO). They were mixed in Ringer solution and diluted (1:9, Merck, Darmstadt, Germany). Diluted samples were spread onto M-Enterococcus agar plates (Difco, Sparks, MD, USA) and incubated at 37 °C for 48 h. Total enterococcal counts were expressed in colony-forming units per mL (cfu/mL). Different colonies were selected, checked for purity and submitted for identification.

### 2.2. Strains Identification

Taxonomic identification was performed using the matrix–assisted laser desorption ionisation time-of-flight spectrometry identification system (MALDI-TOF MS, Bruker Daltonics, Billerica, MD, USA) based on protein “fingerprints“ [19]. Lysates of bacterial cells were prepared according to the producer’s instructions (Bruker Daltonics). Results were evaluated using the MALDI Biotyper 3.0 (Bruker Daltonics, Billerica, USA) identification database. Taxonomic allocation was evaluated on the basis of highly probable species identification (score 2.300–3.000), secure genus identification/probable species identification (2.000–2.299) and probable genus identification (1.700–1.999). Positive controls were those included in the Bruker Daltonics database. Identical colonies evaluated with the same MALDI-TOF score value were excluded. Identified strain ML2/2 was then maintained on M-Enterococcus agar (Difco, Sparks, MD, USA) and stored using the Microbank system (Pro-Lab Diagnostic, Richmond, British Columbia, Canada) for subsequent testing. To exclude/confirm any beneficial character of the ML2/2 strain, metabolic enzyme production, antibiotic susceptibility profile, biofilm formation, oxgall bile-tolerance and low pH-tolerance were tested. In addition, its growth in skim milk and especially its bacteriocin activity were investigated. Basic characteristics such as hemolysis, deoxyribonuclease (DNase) and gelatinase phenotypes were tested as well.

### 2.3. Enzyme Production Testing and Antibiotic Susceptibility Profile

Metabolic enzyme production was tested using the commercial semi-quantitative API-ZYM system (BioMérioux, Marcy l’Etoile, France). The enzymes tested followed the manufacturer‘s recommendation: alkaline phosphatase, esterase (C4), esterase lipase (C8), lipase (C14), leucine arylamidase, valine arylamidase, cystine arylamidase, trypsin, α-chymotrypsin, acid phosphatase, naftol-AS-BI-phosphohydrolase, α-galactosidase, β-galactosidase, β-glucuronidase, α-glucosidase, β-glucosidase, *N*-acetyl-β-glucosamonidase, α-manosidase, and α-fucosidase. Inocula (65 µL-microliter) of McFarland standard one suspensions were pipetted into each well of the kit. Enzyme activities were evaluated after 4 h of incubation at 37 °C and after the addition of Zym A and Zym B reagents. Color intensity values from 0 to 5 and their relevant value in nanomoles (nmoL) were assigned for each reaction according to the color chart supplied with the kit.

Following the EFSA rules for producing antibiograms, the CLSI [20] system was applied with antibiotic strips. Eleven E test/antibiotic strips (Oxoid, Basingstoke, Hampshire, United Kingdom; Fluka, Buchs, Switzerland) were used according to supplier‘s recommendation: novobiocin (Nov, 5 µg), ampicillin (AMP, 10 µg), erythromycin, azithromycin (ERY, AZM, 15 µg), streptomycin (S, 25 µg), chloramphenicol, rifampicin, vancomycin, tetracycline, kanamycin (C, RIF, VAN, TC, KAN, 30 µg) and gentamicin (120 µg). Overnight culture (100 µL) of EM ML2/2 strain was spread on Mueller-Hinton agar (Difco, Sparks, MD, USA). Strips were put on agar plates and cultivated at 37 °C for 24 h.

### 2.4. Hemolysis, DNase and Gelatinase Phenotypes

Hemolysis was detected by streaking the cultures onto BH agar (Difco, Sparks, MD, USA) supplemented with 5% defibrinated sheep blood. Plates were incubated at 37 °C for 24–48 h. Presence or absence of clear zones around the colonies was interpreted as α- and β-hemolysis respectively, while γ-hemolysis indicated negative strains [21].

To determine nuclease activity, each strain was inoculated onto the surface of DNase agar (Oxoid, Basingstoke, Hampshire, United Kingdom) and incubated for 24 h at 37 °C. The production of deoxyribonuclease can be evaluated on this medium as colonies producing DNase hydrolyse the deoxyribonucleic acid (DNA) within the medium. After flooding and acidifying the medium with 1 N HCl, the DNA precipitated out; and the medium became turbid with clear zones around DNase-positive colonies.

Gelatinase phenotype was tested according to Kanemitsu et al. [22]. Briefly, EM ML2/2 was cultivated on Todd-Hewitt agar (Becton and Dickinson, Baltimore, MD, USA) enrihed with gelatinase (30 g per L). Plates were incubated at 37 °C for 48 h. After that, the medium surface was overlaid using a solution of 15% HgCl_2_ in 20% HCl. Turbidity with clear zones around colonies indicated gelatinase activity.

### 2.5. Biofilm Formation Ability

Nowadays, biofilm-forming ability in strains is checked for different purposes; either to eliminate this feature and/or to assess it in terms of beneficial strains. Biofilm formation in EM ML2/2 strain was tested using the quantitative plate assay proposed by Chaieb et al. [23] and Slížová et al. [24]. One colony of EM ML2/2 strain grown on Brain-heart-infusion agar overnight at 37 °C (Difco, Sparks, MD, USA) was transferred into 5 mL of Ringer solution (pH 7.0, 0.75% *w*/*v*) to obtain suspension corresponding to 1 × 10^8^ cfu/mL. A 100 µL aliquot from that dilution was transferred into 10 mL of BHI broth (Difco, Sparks, MD, USA). A volume 200 µL of dilution was inoculated into polystyrene microtiter plate wells (Greiner ELISA 12 Well Strips, 350 µL, flat bottom, Frickenhausen GmbH, Frickenhausen, Germany) and incubated for 24 h at 37 °C. The biofilm formed in the microtiter plate wells was washed twice with 200 µL of deionized water and dried at 25 °C for 40 min. The remaining attached bacteria were stained for 30 min at 25 °C with 200 µL 0.1% (*m*/*v*) crystal violet in deionized water. The dye solution was aspirated away, and the wells were washed twice with 200 µL deionized water. After removal of the water, the plate was dried for 30 min at 25 °C, and the dye bound to the adherent biofilm was extracted with 200 µL 95% ethanol. A 150 µL aliquot was transferred from each well into a new microplate well for optical density (absorbance, A) at 570 nm using an Apollo 11 Absorbance Microplate reader LB 913 (Apollo, Berthold Technologies, Oak Ridge, TN, USA). Each strain and condition was tested in two independent tests with 12 replicates. Sterile BHI was included in each analysis as negative control. *Streptococcus equi* subsp. *zooepidemicus* CCM 7316 was used as positive control (kindly provided by Dr. Eva Styková, University of Veterinary Medicine and Pharmacy, Košice, Slovakia). Biofilm formation was classified as highly positive (A_570_ ≥ 1.0), low-grade positive (0.1 ≤ A_570_ < 1.0) or negative (A_570_ < 0.1) according to Chaieb et al. [23] and Slížová et al. [24].

### 2.6. Tolerance to Oxgall Bile, Low pH and Growth Ability in Skim Milk

Tolerance of EM ML2/2 strain in a bile environment was checked using MRS broth (Merck, Darmstadt, Germany) enriched with 3% and 5% oxgall-bile (Difco, Sparks, MD, USA) according to Gilliland and Walker [25]. Overnight culture of the strain was inoculated (0.1%) into MRS broth without and with oxgall/bile and incubated at 37 °C for 24 h. Viable cells of the tested strain were counted at 0 h and after 24 h incubation of the appropriate dilution (in Ringer solution, Merck) plated on BHA agar (Oxoid, Basingstoke, Hampshire, United Kingdom). Results represent counts of surviving cells of EM ML2/2 strain at 0 h and after 24 h expressed in cfu/mL.

Tolerance to pH 2 and pH 3.0 was tested according to Arboleya et al. [26]. Tubes with MRS broth (Merck) were inoculated with 0.1% culture of EM ML2/2 strain, cultivated overnight at 37 °C, and surviving cells counts (absorbance, A 600) were performed at 0 min (before cultivation) and after 90 min. After A 600 measuring and plating (BHA, Difco, Sparks, MD, USA), cells counts were calculated and expressed in cfu/mL.

Skim milk (Difco, Sparks, MD, USA) in tubes was inoculated with 0.1% culture of EM ML2/2 strain, cultivated overnight at 37 °C. Growth of the strain (absorbance, A 600) was measured at 0 h (before cultivation) and 24 h (end of cultivation). After A 600 measuring and plating (MRS agar), growing cell counts were calculated, compared and expressed in cfu/mL.

### 2.7. Bacteriocin Activity

A qualitative test according to Skalka et al. [27] was applied to detect bacteriocin activity in evaluated strains. Altogether 78 indicator (Gram-positive) bacteria were used; 22 strains of the species *Enterococcus hirae* including faecal strains from ostriches (7), pheasants (5), dogs (9) and one strain from dairy product. In addition, 40 *E. faecium* strains and three *E. faecalis* strains from different dairy products (cheeses, milk and yogurt) were used as indicators. Moreover, 12 various staphylococcal species strains (*Staphylococcus capitis*, *S. xylosus*, *S. equorum*, *S. sciuri*, *S. vitulinus*, *S. hominis*, *S. epidermidis*) isolated from goat milk and the principal (most susceptible) indicator strain *E. avium* EA5 (isolated from piglets’ faeces) were also used as indicators. All indicators were isolated in our laboratory. BHagar (1.5%, *v*/*w*) was used for the bottom layer and 0.7% (*v*/*w* BHA) enriched with 200 µL of 18-h culture of the indicator strain with absorbance (A 600) of up to 0.800 was used for the overlay.

Later, concentrated samples of EM ML2/2 strain were prepared in 210 mL of MRS (Merck, Darmstadt, Germany). They were grown overnight at 37 °C (A600, −0.928), then centrifuged 30 min at 10,000× *g*. Supernatants were divided into 3 × 70 mL and pH was adjusted to values 4.5, 6.3 and 7.5. They were treated with EDTA III to neutralize the substance and they were concentrated using Concentrator Plus (Eppendorf AG, Hamburg, Germany) at 45 °C to reach minimal volume (1.5 mL). Inhibition activity of the concentrated substance was tested against EA5 strain using quantitative agar spot testing [28]. Inhibition activity was expressed in Arbitrary unit per milliliter (AU/mL) indicating the reciprocal of the highest twofold dilution of concentrated bacteriocin substances demonstrating complete growth inhibition of the indicator strain.

### 2.8. Preparation of Semi–Purified Substance EM ML2/2 and Its Storage Stability

Finally, semi-purified bacteriocin substances were prepared. EM ML2/2 strain was grown in 210 mL of MRS (Merck, Germany) and incubated overnight at 37 °C (A 600 −0.928). Then broth cultures were centrifuged at 10,000× *g* for 30 min. The pH of the supernatants was adjusted to values 4.5, 6.3 and 7.5; then they were treated with EDTA III and heated at 80 °C for 10 min to neutralize the substance. After that, each volume of supernatant with different pH was precipitated using ammonium sulphate (40% saturation) at 4 °C for 4 h. Precipitated substance was dissolved in minimum (4 mL) volume of 10 mM phosphate buffer (pH 6.5), and its inhibition activity was tested using agar spot testing [28] against EA5 strain. BHA agar (1.5%, *v*/*w*) was used for the bottom layer and 0.7% (*v*/*w* BHA) enriched with 200 µL of an 18-h culture of the indicator strain with absorbance (A 600) of up to 0.800 was used for the overlay. Dilutions of bacteriocin, doses of 10 µL, 1:1 ratio in phosphate buffer (pH 6.5) were aliquoted onto the surface of soft agar. The plates were incubated at 37 °C for 18 h. Clear inhibition zones around the drop dose of bacteriocin was checked and the antimicrobial activity was expressed as AU/mL. The growth of EA5 was inhibited by bacteriocin activity of 3200 AU/mL (pH 6.3). Moreover, 26 different staphylococci were used as indicator strains (those from goat milk and ewe raw milk lump cheeses) but also faecal strains from beavers and horses. Storage stability of the substances was checked after a period of three months storing at −20 °C and inhibition activity was checked against the EA5 strain (AU/mL).

## 3. Results

### 3.1. Strain Identification and Enzyme Production

Total count of enterococci detected in raw goat milk was 1.82 ± 0.5 (log10) cfu/mL on average. Taxonomic allocation of EM ML2/2 strain was based on the MALDI-TOF identification system evaluation with repeated score value 1. 939 indicating probable genus identification (1.700–1.999). However, comparison with the database evaluated *E. mundtii* species with a similar score (Bruker and Daltonics database). Moreover, all phenotypic properties were in accordance with the species *E. mundtii* (data not shown).

*E. mundtii* EM ML 2/2 did not produce any enzyme, or was evaluated in terms of intermediate reaction corresponding to low production of enzyme (5–10 nmoL). Production of the following enzymes alkalic phosphatase, lipase, leucine arylamidase, valin arylamidase, cystine arylamidase, α-chymotrypsin, α-galactosidase, β-galactosidase, β-glucuronidase, α-glucosidase, β-glucosidase, *N*-acetyl-β-glucosaminidase, α-manosidase and α-fucosidase reached 5 nmoL. Esterase and esterase-lipase, trypsin and acidic phosphatase produced low amounts of enzyme at 10 nmoL. EM ML2/2 strain produced 20 nmoL only in the case of enzyme naftol-AS-BI-phosphohydrolase.

### 3.2. Antibiotic Susceptibility Profile, Biofilm Formation and some Virulence factors

EM ML2/2 strain phenotype proved susceptible to vancomycin (MIC = 0.06 µg), AZM (MIC = 12 µg), NOV (0.06 µg), TC (4 µg), ERY (0.5 µg), AMP (0.06 µg), C and GM (12 µg); i.e., to clinically-important antibiotics. However, it was resistant to streptomycin (256 µg), RIF (256 µg) and KAN (128 µg); which is obligatory in some enterococci because of their chromozomally coding marker. EM ML2/2 did not form biofilm (0.075 ± 0.02); it was hemolysis negative (ᵧ-hemolysis), DNase-negative and showed gelatinase-negative phenotype. The last three properties were checked to eliminate its pathogenic potential.

### 3.3. Tolerance to Oxgall Bile, Low pH, Growth in Skim Milk

*E. mundtii* EM ML2/2 showed high survival ability in 3% and 5% medium with oxgall/bile which indicates its tolerance of salts. 88% and 96% of the cell count was read after 24 h cultivation in medium enriched with salt compared with bile medium respectively at the start of cultivation (0 h, 5.84 ± 0.08 cfu/mL: 24 h, 5.00 ± 0.09 cfu/mL; (0 h, 5.70 ± 0.11 cfu/mL: 24 h, 5.47 ± 0.00 cfu/mL).

The surviving cell count for EM ML2/2 was 1.50 ± 0.50 cfu/mL after 90 min at pH 2, compared to 0 min (6.12 ± 0.32 cfu/mL). In medium with pH 3 higher survival ability of EM ML2/2 strain was noted (2.15 ± 1.15 cfu/mL) after 90 min compared to minute 0 (6.55 ± 0.20 cfu/mL). Survival (20%) of EM ML2/2 strain was checked after 24 h cultivation in skim milk compared to 0 min (5.60 ± 0.02 cfu/mL: 1.15 ± 0.15 cfu/mL).

### 3.4. Bacteriocin Activity

Out of 78 different Gram-positive indicator strains used, first the growth of 28 (36%) was inhibited, including several enterococcal species; however, 12 different staphylococci from goat milk were not inhibited (Table 1). Growth of fecal strains *E. hirae* from ostriches and pheasants as well as *E. hirae* EHDak (from yogurt) was inhibited, with an inhibition zone ranging from 30 up to 32 mm (in the case of ostrich‘ strains), 25–37 mm in strains from pheasants, and 25 mm for E. *hirae* EHDak strain (Table 1). Faecal canine *E. hirae* strains were less inhibited (6 out of 9 strains) with inhibition zone size ranging from 25 to 28 mm. When 40 *E. faecium* strains were used as indicators, growth of only seven strains was inhibited (17%) with zone size in the range 10–37 mm. Two out of three *E. faecalis* were inhibited (22–24 mm, Table 1). Staphylococci from goat milk were not inhibited. Finally, the principal indicator strain *E. avium* EA5 (from piglet faeces) was inhibited with zone size 25 mm. Although seven out of 40 *E. faecium* indicators were inhibited, the highest activity was reached against *E. faecium* EF6R from Romadour cheese (37 mm). The lowest was inhibition activity against *E. faecium* EFK6 (10 mm) from “korbáčik“, a traditional smoked cheese product made from ewe milk. Although up to now Gram-negative bacteria were not used as indicators in this testing, EM ML2/2 strain was recognized as having with inhibition activity, and concentrates were therefore prepared.

Concentrates with different pH of substance produced by *E. mundtii* EM ML2/2 showed antimicrobial activity; however, concentrate with pH 6.3 reached the highest inhibition activity (1600 AU/mL, Table 2) in agar spot testing against EA5 strain. Concentrates with pH 4.5 and pH 7.5 showed inhibition activity as well (200 AU/mL). These substances were stored for one month at −20 °C and then their remaining activity was checked using agar spot tests. Although their activity was decreased, the highest activity was still detected in concentrate with pH 6.3 (400 AU/mL).

### 3.5. Preparation of Semi–Purified Substance EM ML2/2 and Its Storage Stability

Semi-purified substances (SPS) with pH 4.5 and 7.5 were not active; but SPS with pH 6.3 produced inhibition activity of 3200 AU/mL (Table 2). This substance appears not to remain stable for long under −20 °C storage conditions; when its activity after storage was tested, it decreased to 100 AU/mL after a period of three months (Table 2). Semi-purified substance was used to treat 26 strains of different staphylococci from goat milk, ewe raw milk lump cheese and from faeces of animals. Out of 9 strains from goat milk, two strains were inhibited (*Staphylococcus sciuri* Sci 32/1 and *S equorum* Sq40/2 (100 AU/mL); the other strains (*S. capitis* Sca 11/2, *S. sciuri* Sci 52/1, *S. vitulinus* Sv 38/1, *S. xylosus* SX 49/1, SX 50/1, *S. hominis* SHo 41/2 and *S. epidermidis* SE 30) were not inhibited. Among 9 staphylococci from cheeses, two strains were inhibited (*S. xylosus* SXOS 7/2, *S. simulans* SmiOS 17/6, 100 AU/mL). The strains *S. xylosus* SXOS2/3, *S. simulans* SmiOS14/1, *S. sciuri* SciOS5/1, SciOS18/1, SciOS8/1, SciOS17/4 and SciOS6/3) were not inhibited. Two faecal canine staphylococci tested were inhibited-*S. warneri* SWBado (200 AU/mL) and *S. aureus* SADarty (400 AU/mL). Out of 3 staphylococci from beavers (*S. cohnii* Sco131, *S. epidermidis* SE89 and *S. haemolyticus* SHae 61), the growth of last one strain was inhibited (100 AU/mL). Three fecal horses staphylococci were tested (*S. capitis* Sca 5PL, *S. epidermidis* 2PL/2 and SEK/2PL) and two strains were inhibited (SEK/2PL and SE2PL/2, 100 AU/mL). Altogether 9 out of 26 strains were inhibited with SPS (35%).

## 4. Discussion

There is no information regarding the total enterococcal count in goat milk. In general, their counts are more often involved in the total LAB amount. Goat milk can contain enterococci because of its processing and the handling of animals. The count detected in this study (1.82 ± 0.5 cfu/mL) is low, giving the opportunity for both responses, either to select beneficial strain or to exclude those microbiota using bacteriocins, for example, or their producers [5].

Going through the literature, it seems that more studies present *E. mundtii* detection in association with fish [16,17,29]. However, in our previous studies, *E. mundtii* was a frequent inhabitant of the horses gut, for example [30,31]. *E. mundtii* was first described in 1986 by Collins et al. [32], who provided differentiation of three similar species; *E. casseliflavus*, *E. gallinarum* and *E. mundtii*. Although, MALDI-TOF mass spectrometry has some limitations e.g., its database spectrum, it represents an appropriate method for microbial identification [33]. For EM ML2/2 to be used in practice, confirmation using PCR would have to be added at least, and also sequencing. This would be especially focused on its bacteriocin genome sequencing. In Italy, the island of Sardinia is a major goat milk producing region; *E. faecium* species were mostly genotyped in goat milk [34]. Our detection of *E. mundtii* from raw goat milk with bacteriocinogenic potential is an original result. EM ML2/2 strains did not produce any damaging enzymes. In enterococci, endo-β-*N*-acetylglucosaminidase is the enzyme which is required for them to proliferate *in vivo.* This enzyme cleaves mannose-type glycans in glycoproteins between the *N*-acetylglucosamine residues of the pentasaccharide core [35]. EM ML2/2 produces low amounts (5 nmoL) of this enzyme. However, beneficial enzyme β-galactosidase is also produced in the same low amount. That particular lactase is widely used in the dairy industry for production of lactose –free milk for consumption by lactose intolerant-people [36].

DNase and hemolysis (ᵧ-hemolysis) were negative in *E. mundtii* EM ML2/2. DNase and hemolysis are considered to be virulence factors. DNase catalyses the degradation of DNA making it the subject of a routine diagnostic test which is typical mainly for *S. aureus* strains. Knowing that *E. mundtii* EM ML2/2 is DNase negative and hemolysis-negative is therefore a promising development regarding its application ability after eliminating its pathogenic character.

Gelatinase is one of the virulence factors appearing especially in *E. faecalis*. It is a proteolytic enzyme allowings a living organism to hydrolyse gelatin into sub-components (polypeptides, peptides, and amino acids) which can cross the cell membrane and be used by the organism. It is also possible to detect the *GelE* gene using PCR, which can be confirmed or eliminated with phenotype testing. To our knowledge, in *E. faecium* HC73 strain from beavers for example, negative gelatin phenotype was associated with no *GelE* gene detection [37].

Enterococci can feature both natural (intrinsic) and acquired (transferable) resistance [38]. However, EM ML2/2 strain showed a phenotype susceptible to clinically-important antibiotics.

Due to their frequent salt and acid-tolerance, enterococci are highly adaptable to different food systems [1]. In our study, *E. mundtii* EM ML2/2 tolerated bile and low pH. Moreover, it grew well in skim milk; so this characteristic could be used for its technological testing for use in dairy production for instance.

In clinical strains of enterococci, biofilm production is fundamental in causing diseases [1]. However, as mentioned previously, biofilm formation can also be associated with beneficial strains [39]. Although EM ML2/2 appears to be beneficial, it was non-biofilm-forming, which may be understood at this moment as its positive property.

Although Listeriae were not involved as indicators in testing here, mundticin L for example produced by *E. mundtii* CUGF08 from alfalfa sprouts belonging in Class IIa of enterocins, showed anti-listeria activity [17]. Campos et al. [29] reported a bacteriocin from *E. mundtii* isolated from turbot. That particular bacteriocin was active in pH range 3.5–6.5 and inhibited *S. aureus* as well. Although these are preliminary results, the bacteriocin substance presented in this study seems to be involved in Class II. enterocins; further testing is in progress. It appears that relatively few studies have been published regarding the *E. mundtii* bacteriocins; those producer strains were mostly isolated from fish and their purposes was application in fish processing. Our strain originated from raw goat milk, and we had not previously found that particular source strain producing any bacteriocin, which means that this is an original contribution to mundticin studies and the field of their application. Chikindas et al. [40] reported on this area of bacteriocin research as exciting, potentially leading to new inventions and new applications through possibilities of new progressive sequencing techniques for studying bacteriocins. In spite of the fact that enterococci still appear to be controvers bacteria from the safety point of view, they have real potential as probiotic/beneficial additives, especially because of their bacteriocins. Moreover, EFSA regulation [13,14] recommend assessing each and every candidate enterococcal strain according to all the criteria required for identifying beneficial strains. In any case, knowing bacteriocin potential of enterococcal strains originating from raw goat milk is an original contribution which might stimulate further research in the future.

## 5. Conclusions

*Enterococcus mundtii* EM ML2/2 isolated from Slovak goat milk produces a bacteriocin substance. This antimicrobial substance inhibited growth in up to 36% of various indicator strains respectively in our study; the highest inhibition activity of 1600 AU/mL was demonstrated using concentrated bacteriocin substance EM ML2/2 with pH 6.3. Semi-purified substance produced inhibition activity of 3200 AU/mL. Based on these results, in the future EM ML2/2 strain and/or its bacteriocin substance could be used on farms for bioprotection during first treatment in milk production; that is after safety assessment of the EM ML2/2 strain, of course. The bacteriocin potential of *E. mundtii* strain from Slovak raw goat milk species has not been found previously; so, this is an original contribution and it may open up space for future research.

## Figures and Tables

**Table 1:** Inhibition activity of *Enterococcus mundtii* EM ML2/2 substance tested by Skalka method (1983).

Indicators	*Enterococcus mundtii* EM ML2/2	
	Number of Strains Tested/Number of Strains Inhibited	Inhibition Zones in mm
*Enterococcus hirae*	7/7	30–32
*E. hirae*	5/5	25–37
*E. hirae*	9/6	25–28
*E. faecium*	40/7	10–37
*E. faecalis*	3/2	22–24
*E. hirae*	1/1	25
*E. avium* EA5	1/1	25
*Staphylococci*	12/0	0

78 indicator strains; *Enterococcus hirae* from faeces of ostriches (7), *E. hirae* from faeces of pheasants (5), *E. hirae* from faeces of dogs (9), *E. faecium* from 40 different dairy products (milks, cheeses, yogurts), *E. faecalis* from different dairy products (3), *E. hirae* from different dairy products (1), various species of staphylococci (12).

**Table 2:** Inhibition activity of concentrates, semi-purified substance EM ML2/2 and their stability expressed in Arbitrary units per mL.

EM ML2/2	pH 4.5	pH 6.3	pH 7.5
Concentrate	200	1600	200
Stability one month	200	400	200
Semi-puri	x	3200	x
Stability one month	x	1600	x
Two months	x	400	x
Three months	x	100	x

x, it means that those pH were not active.
